# Retinal Vessel Density and Treatment Intensity among Adults with Retinal Vein Occlusion: A Swept-Source Optical Coherence Tomography Angiography Study

**DOI:** 10.3390/jcm11102892

**Published:** 2022-05-20

**Authors:** Brian T. Cheng, Shubhendu Mishra, John M. Bryan, Saena A. Sadiq, Nathan C. Sklar, Emily G. Suen, Taha O. Mohammed, Rukhsana G. Mirza

**Affiliations:** Department of Ophthalmology, Northwestern University Feinberg School of Medicine, Chicago, IL 60611, USA; brian.cheng@northwestern.edu (B.T.C.); shubhendu.mishra@northwestern.edu (S.M.); john.bryan@northwestern.edu (J.M.B.); saena.sadiq@northwestern.edu (S.A.S.); nathan.sklar@northwestern.edu (N.C.S.); emily.suen@northwestern.edu (E.G.S.); taha.osman@northwestern.edu (T.O.M.)

**Keywords:** retinal vein occlusion, optical coherence tomography angiography, treatment response, imaging

## Abstract

Previous studies have shown retinal vein occlusion (RVO) is associated with changes in vessel density visible on swept-source optical coherence tomography angiography (ss-OCTA). This study aimed to characterize retinal changes on ss-OCTA among RVO patients stratified by the need for continuous anti-VEGF therapy. This cross-sectional study of 24 RVO patients ≥ 18 years were imaged with SS-OCT-A. Patients were categorized into continuous vs. limited therapy (≥1 vs. no injections in previous 12 months) based on recurrence of intraretinal fluid (IRF) on OCT. Images were analyzed using ImageJ. T-tests were used to compare vessel density of the macula and peripheral retina. Overall, RVO patients undergoing continuous therapy (*n* = 14) had higher diabetes prevalence, worse baseline visual acuity, and higher baseline macular thickness compared to the limited (*n* = 10) therapy group. Continuous therapy was associated with lower macular VD in the combined retina layer and the superficial capillary plexus (SCP), but not in the deep capillary plexus (DCP). Further, the continuous therapy group exhibited lower peripheral VD in the combined retina layer, and no difference in the SCP and DCP layers when analyzed separately. In conclusion, RVO patients requiring continuous anti-VEGF injections demonstrate reduced VD of the macula and in the periphery on SS-OCTA imaging. SS-OCTA may be valuable for monitoring and prognosticating treatment for RVO patients.

## 1. Introduction

Retinal vein occlusion (RVO) is the second most common vascular disease of the retina and can occur as either central (CRVO) or branch retinal vein (BRVO) patterns [1]. The resulting cystoid macular edema (CME) due to breakdown of the blood-retinal barrier may cause vision impairment [2,3]. Anti-VEGF agents are the mainstay of treatment for CME secondary to RVO [4,5,6,7]. RVOs can be assessed by fluorescein angiography (FA), but FA is suboptimal for serial evaluation due to various risks, including the infrequent but serious risk of anaphylaxis [8,9,10].

Optical coherence tomography angiography (OCTA) is used to visualize the microvasculature of the retina and choroid. With greater definition than dye angiography [11,12], swept-source OCTA (ss-OCTA) enables widefield imaging of the retina. This is particularly useful in evaluating RVO, as capillary abnormalities often extend beyond the macula. Previous studies have shown that ss-OCTA can reliably detect peripheral retina nonperfusion and ischemic changes in patients with RVO [13,14].

Previous studies using ultra widefield FA found that a greater extent of peripheral ischemia drives persistent macular edema and potentially a continuous need for anti-VEGF injections among patients with diabetic retinopathy [15]. Previous research has shown that ss-OCTA can be used to evaluate ischemic changes in RVO patients [11,13,14], the relationship of changes in vessel density (VD) with the need for continuous anti-VEGF therapy in patients with RVO is not well-understood. In this study, we assessed the peripheral and macular VD among patients who did versus did not require continuous anti-VEGF therapy to explore the association of ss-OCTA parameters and treatment burden. Understanding the clinical applications of ss-OCTA parameters is important to the development of future artificial intelligence algorithms and optimal management strategies of RVO.

## 2. Materials and Methods

### 2.1. Study Design and Patient Cohort

We conducted a prospective cross-sectional study of patients receiving management of unilateral CRVO or BRVO at Northwestern Memorial Hospital between July 2020 and February 2021. Eligible patients were adults (≥18 years) with a clinical diagnosis of RVO. Exclusion criteria were patients with confounding ophthalmic disease, obstructed fundus view, or inability to obtain good quality images. Informed consent was obtained before participation. Study protocol and design were approved by the Northwestern University Institutional Review Board and adhered to the tenets of the Declaration of Helsinki. Neither patients nor the public were involved in the design, conduct, reporting, or dissemination plans for this project.

### 2.2. Study Procedure

Data on socio-demographic (age, gender) and clinical characteristics (hypertension, diabetes mellitus, smoking status) and clinical exam (logarithm of the Minimum Angle of Resolution Best-Corrected Visual Acuity [LogMAR BCVA] during initial presentation, LogMAR BCVA on the day of SS-OCTA imaging, macular thickness at presentation, macular thickness on day of imaging) were obtained from chart review. SS-OCTA imaging was obtained using the PLEX Elite 9000 (Carl Zeiss Meditec Inc., Dublin, CA, USA), a device that obtains 100,000 A-scans per second with a capture depth of 3 mm; 3 mm × 3 mm standard and 12 mm × 12 mm wide-field scans centered on the fovea were acquired in each eye of the participants.

### 2.3. Image Analysis

The primary study outcome was vessel density (VD) of the superficial (SCP) and deep capillary plexus (DCP), both in the 3 mm × 3 mm macula and 12 mm × 12 mm wide-field peripheral retina. Patients were stratified into those undergoing continuous (≥1 injection in the 12 months preceding enrollment and ss-OCTA imaging) and limited anti-VEGF therapy (no injections in the prior 12 months due to clinical stability). Patients with limited anti-VEGF therapy may have received injections in the past, but not in the 12 months preceding ss-OCTA imaging. The need for continuous vs. limited anti-VEGF therapy was decided by the patient’s ophthalmologist based on macular edema and OCT findings, and participation in this study did not influence treatment decisions. Images were excluded in the case of poor image quality, segmentation errors, motion artifact, and/or blink artifacts.

Analysis was performed on the SCP, the DCP with projection artifact removed, and the entire retina. These images were obtained as outputs of the Macular Density v0.7.3 algorithm from the Advanced Retina Imaging Network. The images were then analyzed using ImageJ (National Institutes of Health, Bethesda, MD, USA). Images were thresholded and skeletonized using the method described by Lim et al. to remove background contrast for accurate estimates of vessel density [16].

**Figure 1:**
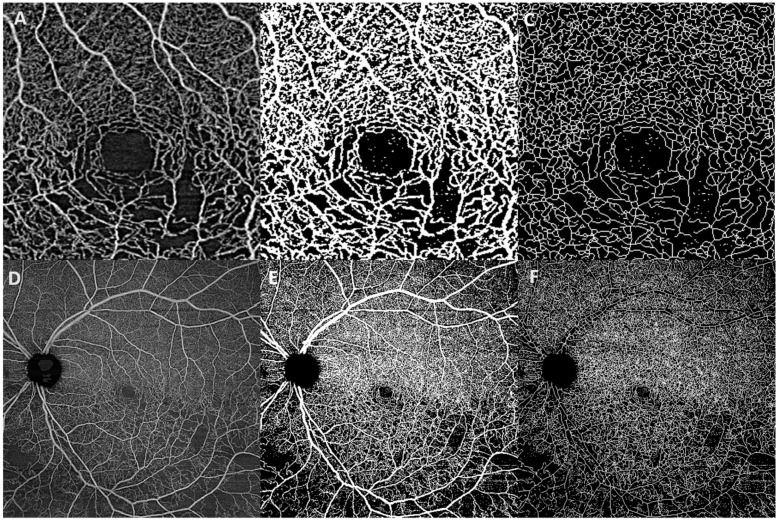
Image analysis of the SS-OCT angiography of the affected eye of a 72-year-old woman with a branch retinal vein occlusion: (**A**) A 3 mm × 3 mm macular image of the retinal layer of the affected eye. (**B**) The 3 mm × 3 mm macular image after the thresholding algorithm is applied. (**C**) The 3 mm × 3 mm skeletonized image of the retinal layer. (**D**) A 12 mm × 12 mm image of the retinal layer of the affected eye. (**E**) The 12 mm × 12 mm image of the retinal layer after the thresholding algorithm is applied. (**F**) The 12 mm × 12 mm skeletonized image of the retinal layer.

Vessel density was calculated from the skeletonized image of the retinal vasculature, subtracting the contribution of the foveal avascular zone (FAZ) (Figure 1). The Macular Density v0.7.3 algorithm determined the size and location of the foveal avascular zone. The vessel density was separately estimated for each of the three layers for each eye. The mid-peripheral vessel density was calculated as a mean of the four quadrants of the retina. All study patients with RVO had unilateral disease. As such, the patient’s contralateral fellow eye without RVO was used as a control group for comparison.

### 2.4. Statistical Analysis

Frequency and prevalence were calculated for categorical variables, and mean and standard deviation were reported for continuous variables. Rao–Scott χ^2^ tests and Student’s *t*-tests were used to assess socio-demographic and clinical differences between eyes with limited vs. continuous anti-VEGF therapy.

We calculated mean VD and standard deviation in the retina, SCP, and DCP. Two-sample *t*-tests were used to examine differences in VD of that undergoing continuous and limited anti-VEGF treatment. We also compared to the patient’s contralateral fellow eye without RVO as controls.

All data maintenance and statistical analyses were conducted in SAS v9.4 (SAS Institute, Cary, NC, USA). A two-sided *p* < 0.05 was considered statistically significant.

## 3. Results

A total of 28 patients were enrolled, and imaging data were obtained. Four patients were excluded due to obstructed fundus view or poor image quality. Of 24 patients with RVO in the final study cohort, 12 were male, mean ± standard deviation age was 64.7 ± 11.9 years, and 14 were receiving continuous anti-VEGF therapy. Compared to those receiving limited therapy, patients with continuous anti-VEGF treatment group had greater rates of diabetes (*p* = 0.003), worse baseline vision at the time of RVO diagnosis (*p* = 0.008), and higher baseline macular thickness at the time of diagnosis (*p* = 0.04) (Table 1). No patients in our study cohort received macular or peripheral retinal laser treatment.

There were no differences between continuous and limited therapy with respect to age, gender, RVO type, hypertension, smoking status, visual acuity, and macular thickness at the time of imaging. The duration of RVO was significantly shorter in eyes receiving continuous therapy, compared to eyes receiving limited anti-VEGF therapy (*p* = 0.04). Yet, eyes with continuous and limited therapy had a similar number of total anti-VEGF injections since their diagnosis (*p* = 0.14). Two patients undergoing continuous treatment were diagnosed with neovascularization, and one patient had proliferative diabetic retinopathy. There were no statistically significant differences in neovascularization or diabetic retinopathy by treatment group.

### 3.1. Macular VD

Continuous therapy was associated with lower macular VD in the combined retinal layers (49.22 vs. 58.56, *p* = 0.002) and the SCP (46.55 vs. 56.00, *p* = 0.001) compared to non-RVO fellow eyes (Table 2). There was no significant difference in the DCP of the macular VD of RVO eyes receiving continuous anti-VEGF injections vs. the fellow eyes (*p* = 0.70). The macular VD was similar, comparing RVO eye with limited therapy with their fellow eye.

As shown in Table 3, continuous injections in RVO eyes were associated with lower macular VD in the combined retina (mean: 49.22 vs. 57.41, *p* = 0.04) and the SCP (46.55 vs. 54.24, *p* = 0.04) compared to RVO eyes with limited treatment; the VD in the DCP (47.20 vs. 47.96, *p* = 0.80) was similar.

### 3.2. Peripheral VD

The peripheral VD in the combined retina layers was lower in RVO eyes receiving continuous therapy than fellow eyes (70.66 vs. 82.22, *p* = 0.02). However, when the SCP (73.99 vs. 76.69, *p* = 0.38) and DCP plexus (92.30 vs. 91.95, *p* = 0.89) layers were analyzed separately, no significant difference in VD was noted (Table 2). No significant differences in peripheral VD were observed comparing RVO eyes receiving limited therapy and their fellow non-disease eyes.

## 4. Discussion

This study found retinal patterns on SS-OCTA that are related to degree of treatment burden in RVO. In particular, our data show that patients who require continuous anti-VEGF injections for CME have decreased vessel density in the macula, compared to patients who require limited therapy and compared to fellow eyes without RVO. These results confirm and expand upon previous studies that have demonstrated macular retinal changes associated with RVO [12,17,18], whereas previous studies focused on characterizing macular changes associated with RVO, we used SS-OCTA to assess if there were also peripheral changes. This study found lower peripheral retina vessel density in the overall retina among eyes with RVO requiring continuous anti-VEGF treatment versus fellow eyes without RVO. Taken together, these findings suggest there are both macular and peripheral retina changes on SS-OCTA among patients with RVO, and these differences are associated with the need for continuous anti-VEGF treatment. These results have important implications for the understanding of non-invasive retinal imaging and its potential ability to improve clinical prognostication and treatment monitoring.

Reduced vessel density in the macula is associated with a need for continuous anti-VEGF injections among patients with RVO. These findings are consistent with previous SS-OCTA studies that showed macular vessel density loss associated with RVO [12,17,18]. This study builds on these findings to note that the reduced macular vessel density was only observed in those patients who required continuous anti-VEGF treatment. Previous research suggests that vessel density may act as a surrogate for retinal perfusion, and ischemia may contribute to decreased vessel density [19]. Ischemic insult is associated with increased VEGF secretion, which previous studies found increase retinal vessel permeability and neovascularization. As such, it may be that greater ischemic changes lead to a larger decrease in vessel density and increased VEGF secretion, thereby necessitating more injections. It may also be that continuous anti-VEGF therapy decreases the formation of new vessels in the retina, explaining the decreased vessel density associated with continuous treatment. Future studies are needed to elucidate the relationship of ischemic injury, vessel density, and anti-VEGF treatment among patients with RVO.

On sensitivity analysis, we found that changes in vessel density were present only in the SCP and not in the DCP. In fact, our data showed no difference in DCP vessel density between the three study groups. This is in contrast to a prior study of RVO-related retinal changes using SS-OCTA which found DCP ischemia was predictive of worse visual symptoms [1,2,11,20]. Another study found that SCP vessel density was associated with recurrent macular edema, which may necessitate repeated anti-VEGF injections [1,21]. This may suggest that decreased vessel density of the DCP could predict worse visual symptoms but not increased need for treatment in RVO. On the other hand, reduced macular VD in the SCP may predispose to macular edema recurrence. This supports the potential utility of SS-OCTA to calculate SCP vessel density as a useful metric in the management of RVO.

Prior research has shown that increased macular thickness secondary to macular edema may contribute to reduced BCVA [1,11]. In our study, patients with RVO who required continuous anti-VEGF therapy were more likely to have worse visual acuity and increased macular thickness at presentation. In this case, treatment burden reflects severity of disease. This further highlights the need for new information and strategies to maximize treatment outcomes in these patients.

Vessel density of the peripheral retina was lower in the overall retina among eyes with RVO vs. fellow eyes without RVO. This is consistent with previous studies that found that CME is associated with peripheral retinal ischemia in RVO [15]. Interestingly, there were no observed differences in the SCP or DCP. There are multiple explanations. This may be explained in part by the SS-OCTA methodology that overlays multiple retinal angiography layers to represent a three-dimensional model. Vessels that overlap between the SCP and DCP may have variable effect on vessel density in each individual layer. This may also be attributable to our limited sample size. Eyes with versus without RVO had numerically lower vessel density in the SCP, consistent with differences that this study found in the macula. Our results provide important data to understand changes to the peripheral retina as a result of RVO. However, additional larger scale studies are needed to refine our understanding of changes to the peripheral retinal vessel density.

In this study, the vessel density of eyes receiving limited therapy were similar to that of fellow eyes without RVO, regardless of location (i.e., macular vs. peripheral) and capillary level. It may be that less severe RVO that leads to mild macular edema and requires only limited anti-VEGF treatment causes minimal to no vessel density reduction. It may also be that although fellow eyes do not have RVO, they may not be entirely unaffected by systemic disease, as patients with unilateral RVO may have systemic vascular pathology the affects the contralateral eye [1,22]. With this caveat in mind, these findings suggest that RVO eyes showing similar VD to fellow eyes via SS-OCTA imaging may be managed more conservatively.

SS-OCT-A can non-invasively and rapidly evaluate capillary non-perfusion at a high resolution, has an excellent safety profile, and yields a comparable performance to fluorescein angiography when assessing for microvascular disease. Therefore, SS-OCTA is becoming more prevalent in ophthalmic clinical practice [1,2,11,13]. This imaging modality allows for the acquisition of longitudinal information and provides valuable data that may be able to guide management and prognosis.

The strengths of this study include the prospective patient enrollment of patients with RVO and the use of SS-OCTA that allows for examination of the peripheral retinal vasculature. However, some limitations merit mention. First, this study had a limited sample size that included 14 patients with continuous and 10 patients with limited anti-VEGF therapy. A larger sample size may allow for more robust conclusions and the detection of subtle differences. Second, the increased prevalence of diabetes among patients requiring ongoing anti-VEGF treatment is a confounding factor for VD analysis, albeit one controlled partially by the comparison to the fellow eye. In addition, the presence of ischemic and neovascular forms of RVO in the ongoing treatment group could also influence VD results. Third, our method for calculating VD was limited by pixels and led to higher values to be estimated in the peripheral retina. Therefore, mean VD values in the peripheral retina on 12 × 12 images are higher than those of the macula on 3 × 3 images. For this reason, we did not make any statistical comparisons of peripheral to macular retina vessel density. Fourth, the calculation of VD required manual outlining of the foveal avascular zone, and there is a risk of human error in tracing the FAZ. Finally, this was a cross-sectional analysis that obtained SS-OCTA in patients at a single point in their clinical care and causation can therefore not be assessed. As such, we were unable to examine changes in vessel density associated with resolution of RVO pathology. Future longitudinal studies are needed to better understand the changes in retinal vasculature over time and determine the effect of anti-VEGF treatments.

## 5. Conclusions

In conclusion, decreased vessel density in the macula and peripheral retina were associated with the need for continuous anti-VEGF intravitreal injections among patients with RVO. This study shows the possible utility of ss-OCTA to monitor disease progression and management of patients with RVO. These findings may help clinicians to interpret retinal imaging, estimate prognosis, and guide patient discussions. It is important to improve the identification of RVO patients with a higher risk profile. This study advances the understanding of how safe, non-invasive retinal imaging may be used to provide in vivo insight of the retinal microcirculation. Future studies will determine the optimal strategies to use ss-OCTA to tailor the clinical care of patients with RVO.

## Figures and Tables

**Table 1:** Baseline characteristics of retinal vein occlusion patients receiving either continuous anti-VEGF therapy or limited therapy.

	All RVO Patients (*n* = 24)	Continuous Anti-VEGF Therapy (*n* = 14)	Limited Therapy (*n* = 10)	*p*-Value
**Age, years**				
μ (SD)	64.7 (±11.9)	64.9 (±12.1)	64.5 (±12.2)	0.94
**Gender**				
Male (%)	12 (50.0)	5 (35.7)	7 (70.0)	0.10
Females (%)	12 (50.0)	9 (64.3)	3 (30.0)	0.81
**Smoking status**				
Never (%)	14 (63.6)	8 (61.5)	6 (66.7)	-
Former (%)	8 (36.4)	5 (38.5)	3 (33.3)	
Systemic hypertension (%)	17 (70.8)	10 (71.4)	7 (70.0)	0.94
Diabetes (%)	8 (33.3)	8 (57.1)	0 (0.0)	**0.003**
**RVO**				
CRVO (%)	13 (54.2)	7 (50.0)	6 (60.0)	0.63
BRVO (%)	11 (45.8)	7 (50.0)	4 (40.0)	
**Baseline LogMAR BCVA**				
μ (SD)	0.37 (±0.26)	0.49 (±0.23)	0.22 (±0.21)	**0.008**
**Baseline MT (μm)**				
μ (SD)	491.6 (±211.3)	573.7 (±202.4)	393.2 (±185.3)	**0.04**
**IOP at imaging**				
μ (SD)	16.6 (±3.5)	16.0 (±3.2)	17.4 (±3.9)	0.36
**LogMAR BCVA at imaging**				
μ (SD)	0.19 (±0.42)	0.29 (±0.54)	0.06 (±0.13)	0.15
**MT at imaging (μm)**				
μ (SD)	249.6 (±87.0)	255.6 (±106.1)	242.4 (±61.5)	0.73
**Duration of RVO (months)**				
μ (SD)	41.3 (±37.5)	27.2 (±31.0)	57.7 (±38.9)	**0.04**
**# of anti-VEGF injections**				
μ (SD)	10.0 (±11.8)	13.5 (±14.5)	5.8 (±6.8)	0.14

Rao–Scott χ^2^ and independent *t*-tests assessed differences by treatment regimen (continuous vs. limited therapy) for categorical and continuous variables, respectively. Boldface text indicates statistical significance *p* < 0.05. anti-VEGF: anti-vascular endothelial growth factor; μ: mean; SD: standard deviation; RVO: retinal vein occlusion; CRVO: central retinal vein occlusion; BRVO: branch retinal vein occlusion; BCVA: best-corrected visual acuity; MT: macular thickness.

**Table 2:** Vessel density in the macula and mid-periphery in the retinal, SCP, and DCP in eyes with retinal vein occlusions.

	Retina	*p*-Value	Superficial Capillary Plexus	*p*-Value	Deep Capillary Plexus	*p*-Value
Macular VD						
Continuous μ (SD)	49.22 (±12.00)	**0.002**	46.55 (±11.28)	**0.001**	47.20 (±9.35)	0.70
Limited μ (SD)	57.41 (±4.88)	0.51	54.24 (±5.24)	0.33	47.96 (±5.46)	0.96
Fellow Eye μ (SD)	58.56 (±4.39)	ref	56.00 (±4.22)	ref	48.03 (±3.27)	ref
Mid-peripheral VD						
Continuous μ (SD)	70.66 (±14.27)	**0.02**	73.99 (±10.94)	0.38	92.30 (±4.29)	0.89
Limited μ (SD)	76.15 (±5.75)	0.13	72.48 (±5.47)	0.07	90.78 (±4.65)	0.66
Fellow Eye μ (SD)	82.22 (±11.28)	ref	76.69 (±5.79)	ref	91.95 (±8.04)	ref

Bivariable *t*-tests were used to assess VD in the affected eye with RVO among patients with continuous and limited therapy, compared to VD of the fellow eye without RVO. Boldface text indicates statistical significance *p* < 0.05. VD: vessel density; μ: mean; SD: standard deviation; ref: reference level.

**Table 3:** Comparison of vessel density among patients receiving continuous vs. limited anti-VEGF therapy for RVO.

	Continuous Therapy	Limited Therapy	*p*-Value
Macular VD			
Retina, μ (SD)	49.22 (±12.00)	57.41 (±4.88)	**0.04**
Superficial capillary plexus, μ (SD)	46.55 (±11.28)	54.24 (±5.24)	**0.04**
Deep capillary plexus, μ (SD)	47.20 (±9.35)	47.96 (±5.46)	0.82
Mid-peripheral VD			
Retina, μ (SD)	70.66 (±14.27)	76.15 (±5.75)	0.22
Superficial capillary plexus, μ (SD)	73.99 (±10.94)	72.48 (±5.47)	0.67
Deep capillary plexus, μ (SD)	92.30 (±4.29)	90.78 (±4.65)	0.34

A Bivariable *t*-test was used to assess differences in VD in the affected eye with RVO among patients with ongoing compared to limited anti-VEGF therapy. Boldface text indicates statistical significance *p* < 0.05. VD: Vessel density; μ: mean; SD: standard deviation.

## Data Availability

Data is available upon reasonable request.
